# What about faculty?

**DOI:** 10.7554/eLife.54551

**Published:** 2020-01-08

**Authors:** Hilal A Lashuel

**Affiliations:** 1 Brain Mind InstituteEcole Polytechnique Fédérale de Lausanne (EPFL)LausanneSwitzerland

**Keywords:** mental health, faculty, professor, academia, stress, tenure

## Abstract

After two heart attacks in three years, an associate professor discusses the challenges of faculty life.

The life of a professor is a constant balancing act where we try to juggle personal and professional responsibilities under the pervasive stress of managing expectations in an often hypercompetitive culture. There is always a fear that we may drop the ball, a sense that if that were to happen, we would be alone and the only one to blame. The system assumes that we should be old enough, experienced enough, and tough enough to withstand all the pressure that comes with the job. Being a faculty member in a university can be one of the most fulfilling career paths, but it has also become one of the most stressful jobs.

## The storms of academia

As young scientists taking on a faculty position, we quickly transition from being a team member to a team leader; from never worrying about securing funding to being overwhelmed with grant deadlines; from managing a single project to planning and guiding the work and careers of several students and post-docs; from worrying about ourselves to being absorbed in worrying about everything except our wellness. The great majority of us have never developed a course or taught classes on our own, yet we are all expected to assume these responsibilities.

Many universities give good support when it comes to teaching, yet most offer very little training or help in project and team management, leadership, mentoring and conflict resolution, let alone mental health awareness and intervention. We are expected to learn everything on the job. In other words, we learn by making mistakes that we – and to some extent our students and staff – directly or indirectly end up paying for. Driven by our passion for science, we keep trying and do get better at it, but very rarely pause to assess: “At what cost?”

In other words, we learn by making mistakes that we – and to some extent our students and staff – directly or indirectly end up paying for.

As the tenure clock starts ticking, stress and anxiety often begin to increase; the stakes become higher, and many begin to struggle with the ambiguity of the tenure criteria and the lack of feedback. The pressure mounts to publish papers in ‘high impact journals’, to secure prestigious grants, go on lecture tours, and fill in all the blanks in our CV. Frustration, disappointment, self-doubt or burnout are all too common throughout this journey.

Even after tenure, the pressure often does not go away. Instead, we simply transition from one type of stress to another: from being anxious about publishing and securing tenure to being worried about funding, deadlines, increased administrative duties, the pressure to secure more prestigious grants and awards, and concerns for our reputation. The hypercompetitive academic culture has ways of always keeping you on your toes. For senior faculty, the definition of success is a constantly moving target that is shaped and reshaped by the achievements of our peers and the expectations of our superiors. After achieving tenure, success becomes much harder to define, especially because the more senior we get, the less feedback we receive. What remains constant, however, is the lack of acknowledgement that faculty may be struggling, and the absence of formal or informal support from our institutions.

We constantly preach that failures provide unique learning opportunities and are the stepping stones for success. But when it comes to our own careers, many of us may feel that failure is never an option or something that we are willing to accept, admit or share. Academia is also not the place where we are likely to get second chances. Our peers may view our failure less as a potential learning experience and more as a sign that we are not fit for academia. In a culture of perfectionism and nearly constant peer pressure, the lines between disappointment and failure become very blurry.

Most of us quickly learn that we must project an image of always being in control and unshaken by all the storms of academia. We feel the need to ‘fake it’, until (hopefully) we make it. In reality we, like our students, frequently experience stress, fear and insecurity as well as anxiety, depression and burn out. As faculty, many believe that admitting we are stressed or going through a mental health crisis would be a mistake; that if we do, no one will see us the same way, and that it may compromise our relationship with our students, our colleagues and our superiors. In the absence of a collegial and supportive culture, and with many professors spending most of their time in their office only surrounded by computers, a faculty position can be emotionally, mentally and physically draining. It should not be this way, and no one should suffer alone.

In reality we, like our students, frequently experience stress, fear and insecurity as well as anxiety, depression and burn out.

## Too high a price to pay

Often, we do not realize until it is too late that poor work-life balance and pretending that we are on top of everything comes at a great cost to our health, wellbeing and our families. Pressure, stress and anxiety frequently translate into sleep deprivation, exhaustion, irritability and isolation, all of which negatively affects our quality of life and our interactions with students and colleagues. Chronic stress is also a major risk factor for developing many psychiatric and cardiovascular disorders: I have come to learn this first hand after suffering two heart attacks during the past three years.

We have to equip new postdocs and faculty with the necessary resources and training to manage their new responsibilities, navigate the pre-tenure period and handle their mental health challenges. I am happy that my university – The Ecole Polytechnique Fédérale de Lausanne (EPFL) – has started to recognize and prioritize more professional training and support for faculty, but more work is still needed. As a community, we need to pause, reflect and work together to systematically assess why faculty, but also students and staff in our universities, experience so much stress and so many mental health problems. This, and normalizing the conversation about mental health, are crucial steps to tackling the mental health crisis in our campuses.

While researching the topic, writing and reflecting on my experiences, I realized that there are many things that I could have done differently; I wish I'd had the courage to admit that I was not a superhuman, to seek help to handle my mental health and the daily struggles to achieve work-life balance. From how I managed my time, job expectations and my wellbeing to how I failed at times to fulfil my responsibilities towards my family, I learnt there is no such thing as suppressing your feelings or hiding your struggles. If you do not deal with them, they linger in your head, mess up your sleep pattern, impact your health, and affect the lives of people around you. I regret not taking the time and initiative to seek the assistance of experts and to follow structured training programs that could have helped me with my new responsibilities and to manage my mental health and support my students and colleagues.

But it is never too late: this is exactly what I am doing now. I have come to terms with the fact that balancing my life with my work means saying “no” more, traveling less, prioritizing my health and family, and giving up on trying to please everyone or do everything that can be done. To make more time for myself, my family, my research and my students and group members, I have decided to start by reducing the size of my team, by giving up certain grants and by further consolidating our research programs. I am now more comfortable opening up and discussing my own difficulties with team members and colleagues. I am also more sensitive to the struggles of those around me, and having them share their mental health challenges has been equally therapeutic. At the community level, I plan to organize a series of lectures and activities in 2020 to help normalize the conversation about mental health on campus. I would like to advocate for joint community-based initiatives that create an environment where people struggling with stress and mental health issues feel supported and are not afraid to be excluded.

I hope this article will help others in academia feel comfortable speaking out about their struggles and mental health challenges, and sharing their thoughts, feelings and experiences. As faculty, we cannot take care of our students if we do not learn how to take care of ourselves.

## Note

This Feature Article is part of a collection on mental health in academia.

